# From Venom to Kidney Injury: A Critical Review of *Daboia siamensis* Envenoming

**DOI:** 10.3390/toxins18090389

**Published:** 2026-09-09

**Authors:** Ren-Chieh Wu, Kuang-Ting Yeh

**Affiliations:** 1 Department of Medical Education, Hualien Tzu Chi Hospital, Buddhist Tzu Chi Medical Foundation, Hualien 970473, Taiwan; rcw@tzuchi.com.tw; 2 Program in Pharmacology and Toxicology, Tzu Chi University, Hualien 970374, Taiwan; 3 Department of Emergency Medicine, Hualien Tzu Chi Hospital, Buddhist Tzu Chi Medical Foundation, Hualien 970473, Taiwan; 4 Department of Orthopedics, Hualien Tzu Chi Hospital, Buddhist Tzu Chi Medical Foundation, Hualien 970473, Taiwan; 5 Institute of Medical Sciences, Tzu Chi University, Hualien 970374, Taiwan; 6 Graduate Institute of Clinical Pharmacy, Tzu Chi University, Hualien 970374, Taiwan; 7 School of Medicine, Tzu Chi University, Hualien 970374, Taiwan

**Keywords:** *Daboia siamensis*, Eastern Russell’s viper, acute kidney injury, phospholipase A2, venom-induced consumption coagulopathy, thrombotic microangiopathy, knowledge gaps, antivenom

## Abstract

Acute kidney injury (AKI) is a severe complication of Eastern Russell’s viper (*Daboia siamensis*) envenoming, but species-specific mechanisms, treatment effects, and long-term outcomes remain uncertain. This review addressed four questions: the human renal evidence specific to *D. siamensis*; venom and host pathways contributing to AKI; conclusions supported for antivenom timing and thrombotic microangiopathy (TMA); and quantifiable long-term kidney risk. PubMed and OpenAlex were searched from inception through 3 August 2026. Species-authenticated human evidence was separated from mixed-species or *D. russelii* studies and from venom-analytical and experimental evidence. Referral cohorts show AKI and dialysis burdens, but heterogeneous ascertainment, definitions, and care preclude pooled incidence. Proteomic and experimental studies support interacting coagulopathic, endothelial, hemodynamic, pigment, inflammatory, and direct renal pathways; no human study quantifies their relative contribution, and no *D. siamensis* study localizes specific venom antigens within renal tissue. Observational data support prompt use of indicated, regionally appropriate antivenom but do not validate a universal two-hour renal-protection threshold. TMA and long-term outcome evidence remain largely indirect. Serial renal and coagulation surveillance is warranted irrespective of whether injury is direct or indirect. Prospective, species-authenticated studies linking venom kinetics and toxin profiles to standardized renal phenotypes and follow-up are required.

## 1. Introduction

Snakebite envenoming remains a neglected tropical disease, causing an estimated 1.8–2.7 million envenomings and 81,410–137,880 deaths each year [1]. Eastern Russell’s viper (*Daboia siamensis*) is distributed across parts of mainland Southeast Asia, southern China, and Taiwan. Systemic envenoming can produce acute kidney injury (AKI), venom-induced consumption coagulopathy (VICC), bleeding, shock, thrombocytopenia, hemolysis, and rhabdomyolysis [2,3,4,5,6]. In Taiwan, *D. siamensis* is one of six medically important venomous snakes but is less frequently reported than several other species [2,7].

Taxonomy, species ascertainment, and geography complicate interpretation. Historical reports used names including *Vipera russelli siamensis*, *Daboia russelii* siamensis, and *Vipera russelii formosensis*, and species assignment has variously relied on a specimen, photograph, venom antigen testing, patient recognition, a clinical syndrome, or regional probability [2,3,5,6,8,9,10,11]. Evidence from the western species *D. russelii* is often cited for comparison, yet venom composition, clinical phenotype, and antivenom performance vary across species and regions [3,4,12,13,14,15]. Proteomic and multi-omic studies also demonstrate regional and ontogenetic variation within *D. siamensis* [3,4,16,17,18].

Clinical and pathological reports describe defibrination, bleeding, capillary leak, hypotension, fibrin deposition, renal ischemia, tubular injury, and glomerular or vascular lesions after Eastern Russell’s viper envenoming [6,13,19,20,21,22,23]. Experimental studies using whole venom, purified Russell’s viper venom factor X activator (RVV-X), phospholipase A2 (PLA2), and metalloproteinase-rich fractions demonstrate renal hemodynamic, tubular, coagulation, oxidative, inflammatory, and apoptotic effects [24,25,26,27,28,29,30]. These observations support investigation of interacting toxin, hemostatic, endothelial, perfusion, pigment, and inflammatory pathways rather than a single-agent explanation.

The outcome termed AKI is heterogeneous. Reported phenotypes include transient creatinine elevation, oliguric acute tubular injury, dialysis-requiring failure, cortical necrosis, and less commonly glomerular or vascular lesions [5,8,9,10,11,19,21,22,23,31]. A study that records only peak creatinine cannot distinguish these phenotypes, and dialysis use depends on local capacity as well as biological severity. Renal phenotype, timing, species certainty, and treatment context must therefore be retained when evidence is compared.

This critical review asks four questions: what human renal evidence is genuinely specific to *D. siamensis*; which toxin families and systemic responses plausibly contribute to AKI; what can be concluded about antivenom timing and thrombotic microangiopathy (TMA); and whether long-term kidney risk can be quantified. The synthesis separates species-authenticated clinical observations, mixed or indirect human evidence, and venom-analytical or experimental evidence.

## 2. Literature Search and Evidence Assessment

This article was designed as a structured critical narrative review rather than a systematic review or meta-analysis. PubMed and OpenAlex were searched from inception through 3 August 2026. Four prespecified blocks addressed (1) *D. siamensis* renal alterations and treatment evidence, (2) *D. russelii* renal alterations and treatment evidence, (3) long-term renal outcomes after snakebite, and (4) *D. siamensis* venomics, antivenomics, and toxin studies. *D. russelii* evidence was retained only as indirect comparative context when *D. siamensis*-specific data were absent or insufficient; it was not used to estimate species-specific incidence or treatment effects. Historical taxonomic synonyms were included. Exact strategies, query counts, deduplication rules, and a descriptive selection flow are provided in Table S1 and Figure S1.

The PubMed blocks returned 395 records in aggregate and 307 unique records after removal of 88 cross-query duplicates. OpenAlex returned 638 records in aggregate and 517 unique records after removal of 121 cross-query duplicates. Deduplication across sources by PMID, DOI, and normalized title left 534 unique records. Backward reference checking and targeted citation tracking recovered older nomenclature and relevant guidelines. Reports were retained when they provided extractable human clinical, pathological, venom-analytical, in vivo, or ex vivo evidence relevant to renal mechanisms, antivenom, TMA, or renal recovery. Reports were excluded when exposure or outcome was unrelated, no relevant result was extractable, or an overlapping publication added no relevant information. Thirty-six primary reports and six contextual reviews or guidelines informed the synthesis.

Evidence was classified before interpretation. Tier A comprised human *D. siamensis* reports with a specimen, photograph, venom assay, or clearly defined population from its range. Tier B comprised probable *D. siamensis* or mixed-snakebite cohorts in which species-specific results were incompletely separable. Tier C comprised *D. russelii* or other haemotoxic snakebite studies and was treated as indirect comparative evidence because geographic and species differences preclude direct transfer. Tier D comprised *D. siamensis* venom-analytical, in vivo, or ex vivo evidence. Human studies were appraised across species ascertainment, sampling and design, AKI/outcome definition, comparator or confounding control, and follow-up. Experimental studies were appraised for model relevance, controls, allocation or blinding reporting, exposure and treatment timing, and outcome completeness (Tables S2 and S3). No numeric quality score was used; transferability judgments are summarized in Table S4.

For each clinical report, the synthesis extracted country, recruitment setting, design, sample size, species ascertainment, renal definition, coagulation and systemic features, antivenom exposure, kidney replacement therapy, mortality, and follow-up. Venomic and experimental reports were mapped by venom source, toxin or fraction, model, exposure route, treatment timing, renal endpoints, and antivenom or inhibitor tested. Omic studies were used to identify toxin families, relative abundance, regional or ontogenetic variation, and mechanistic candidates; transcript or protein detection was not treated as clinical proof of nephrotoxicity. Overlapping cohorts were identified explicitly and were not treated as independent corroboration.

No pooled estimate was calculated because species certainty, referral pathways, illness severity, study era, AKI definitions, antivenom access, and kidney replacement therapy availability were clinically incompatible. Screening and appraisal were not independently duplicated; the search flow is therefore descriptive and is not presented as a PRISMA flow diagram.

## 3. Human Evidence for AKI After *Daboia siamensis* Envenoming

### 3.1. Contemporary Thailand and Myanmar Referral Cohorts

The strongest contemporary species-focused evidence is a 10-year Thai poison-center study of 185 patients, including 101 definite bites identified by specimen or photograph. AKI occurred in 50.3%, coagulopathy in 89.2%, thrombocytopenia in 31.4%, rhabdomyolysis in 28.1%, and death in 3.8% [5]. These figures describe a selected poison-center population, not community incidence. Retrospective timing, the combination of definite and clinically diagnosed cases, and referral practices limit transportability to Myanmar, Taiwan, Indonesia, or other Thai settings.

A 12-month prospective registry at a major Mandalay hospital enrolled 948 snakebite patients. Among cases with a recorded identification, 85.4% were assigned to Russell’s viper; the registry attributed all AKI and deaths to *D. siamensis*. AKI was recorded in 59.8% of the overall registry and kidney replacement therapy in 23.9% [8]. A companion report focused on 686 presumed Eastern Russell’s viper bites: 488 patients (71%) met its pragmatic AKI definition and 213 (31%) underwent dialysis [9]. These publications describe overlapping data and must not be counted as independent cohorts. Their high estimates are clinically important for tertiary-care planning but may reflect referral of severe cases, variable identification methods, and local treatment capacity.

A prospective Yangon study found AKI in 140 of 258 envenomed patients; 174 snakes were identified and 147 were Russell’s vipers [10]. Its multivariable associations are useful for risk stratification, but the species-mixed model cannot yield a *D. siamensis*-specific effect estimate. Historical Myanmar studies often provide better species context than modern coding but predate KDIGO definitions and contemporary supportive care. Consequently, apparent differences in AKI or dialysis frequency across decades may reflect definitions and access as much as venom biology.

Across these cohorts, reported variables associated with AKI or dialysis included coagulopathy, thrombocytopenia, shock, rhabdomyolysis, hyponatremia, hematuria, and delayed antivenom [5,10]. The studies used nonrandomized observational designs and adjusted for limited recorded covariates. Dialysis indications and access were not standardized across countries. These results are presented as prognostic associations rather than estimates of causal mediation.

### 3.2. Historical and Taiwanese Evidence

Taiwanese reports demonstrate severity despite the species’ lower frequency. An 18-patient retrospective series described renal failure, defibrination, bleeding, hemolysis, and systemic thrombosis, without the prominent neuromuscular syndrome reported in some *D. russelii* populations [2]. A later 13-patient series measured serum venom and documented early coagulopathy and renal dysfunction [11]. Both series are small and historical, with selected referral cases and changing antivenom availability. They support the possibility of severe renal involvement but cannot establish current Taiwanese incidence or a stable cross-species phenotype.

### 3.3. Synthesis of the Human Burden

Across hospitalized and referral populations, AKI was frequent among patients with systemic *D. siamensis* envenoming, and severe cases required kidney replacement therapy [5,6,8,9,10,11,13,19,20,21,22,23]. No single incidence, dialysis, or mortality percentage is transportable across endemic regions. A 2026 snakebite-AKI meta-analysis reported marked heterogeneity across species and settings [31]. The species-relevant human clinical and pathological evidence is summarized in Table 1.

## 4. Venom Composition and Relevance to Renal Injury

*D. siamensis* venom is a mixture rather than a single nephrotoxin. In Guangxi and Taiwan proteomes, PLA2 represented 22.2% and 24.5% of total venom protein, respectively, while Kunitz-type inhibitors represented 23.2% and 28.2% [3]. In Thai and Indonesian venoms, PLA2 accounted for 37.92% and 48.37%; Kunitz-type inhibitors comprised 22.4% in the Thai venom but were negligible in the Indonesian sample [4]. Snake venom serine proteases (SVSPs) accounted for approximately 13–23%, whereas snake venom metalloproteinases (SVMPs) represented approximately 2–3% in those two proteomes [4]. Because analytical pipelines and pooled venom sources differed, percentages should be interpreted within each study rather than directly combined.

Integrated genomics, venom-gland transcriptomics, and proteomics reveal a broader repertoire. Multi-omic analysis identified toxin isoforms and lower-abundance components, including hyaluronidase, phospholipase B, and waprin [16]. A Myanmar transcriptome described 23 toxin-gene families and 53 full-length toxin transcripts, including C-type lectins, Kunitz inhibitors, disintegrins, bradykinin-potentiating peptide/C-type natriuretic peptide precursors, and factor X activator components [17]. These datasets establish composition and generate testable candidates; transcript abundance is not equivalent to secreted protein dose or renal toxicity.

Individual-component experiments provide functional evidence. Myanmar venom-gland transcriptomics identified P-III SVMPs and factor X activator-related sequences [32]. Purified RVV-X altered renal hemodynamics and coagulation in rats [26], while purified PLA2 and PLA2- or SVMP-rich fractions produced functional or structural renal effects in rabbit in vivo and ex vivo models [25,27,29]. SVMPs can disrupt basement membrane or endothelium and promote hemorrhage, while PLA2 can affect membranes, inflammation, muscle, red cells, and vascular responses. No purified SVSP or Kunitz component has yet been shown to produce a defined renal phenotype in a *D. siamensis*-specific model.

Relative abundance should not be equated with clinical dominance. A low-abundance enzyme may have high catalytic activity, whereas an abundant protein may be weakly toxic or act mainly as a modulator. Proteomic spectral abundance, transcript counts, enzyme assays, immunoblot recognition, murine lethality, and renal injury are distinct endpoints. Table 2 therefore reports the model and evidence boundary for each family rather than assigning a percentage of human AKI to any toxin.

Ontogeny adds another layer. Juvenile *D. siamensis* venom contained higher PLA2, SVMP, and SVSP levels in one experimental series and produced more prominent tubulonephrosis in rabbits, whereas subadult and adult venoms produced different cardiovascular effects and contained relatively more of several other protein families [18]. This is preclinical evidence from pooled venom and does not prove age-dependent human AKI risk. It does, however, show why species name alone is an incomplete exposure definition.

Antivenomics also has mechanistic relevance. Taiwanese monospecific antivenom neutralized major experimental effects of Taiwanese and Guangxi venoms, whereas heterologous products showed weaker performance [3]. Thai monospecific antivenom showed substantial immunoreactivity and experimental neutralization against Thai and Indonesian *D. siamensis* venoms, although potency varied and in vitro or murine endpoints cannot guarantee renal protection in patients [4,12]. Species identification remains essential for selecting the indicated antivenom; within a confirmed species, venom origin and product-specific neutralization also influence expected performance.

## 5. Mechanistic Synthesis

**Figure 1:**
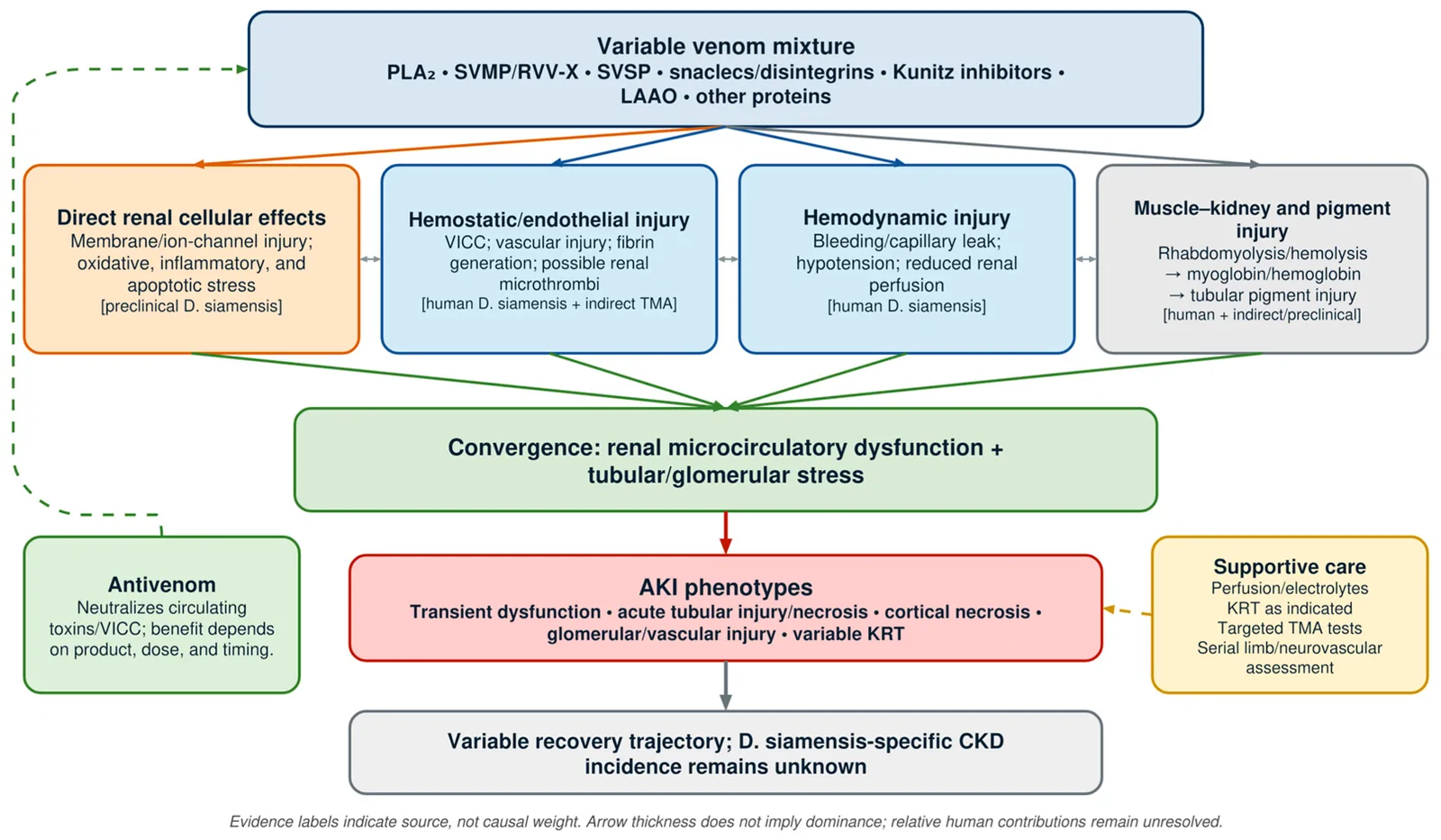
Functional toxin pathways and multifactorial kidney injury after *Daboia siamensis* envenoming. The non-hierarchical scheme shows interacting cellular/tubular, hemostatic/endothelial, perfusion, and muscle/hemolysis pathways. Evidence labels indicate source and transferability, not causal weight; arrow thickness does not imply pathway dominance. Solid arrows show proposed pathophysiological links, gray bidirectional arrows show pathway interactions, and dashed arrows show therapeutic intervention points; colors are used only for visual distinction. Clinical actions are evidence-informed and do not constitute a validated species-specific treatment protocol.

Figure 1 presents a non-hierarchical model in which circulating toxins and host responses converge on renal microcirculatory dysfunction and tubular, glomerular, or cortical injury. The evidence labels identify the source of support, not the percentage of human AKI attributable to each pathway. Table 2 links major toxin families to plausible renal effects and explicitly states the translational limitations.

### 5.1. Hemostatic, Endothelial, and Microvascular Injury

Venom-induced consumption coagulopathy (VICC) is a prominent syndrome after *D. siamensis* envenoming. In 123 proved Burmese Russell’s viper bites, systemic envenoming included incoagulable blood, bleeding, hypotension, capillary leak, and oliguria [6]. Fatal case pathology showed focal hemorrhage, fibrin deposition, acute tubular necrosis, and shock-related injury [19]. In 20 proven bites with severe defibrination, urinary fibrin(-ogen) degradation products were higher among the 10 patients who developed renal failure [20]. These observations support a hemostatic and microvascular contribution, but none isolates it from shock, tissue injury, or simultaneous exposure of the kidney to venom.

Detailed factor kinetics are best characterized in prospective *D. russelii* studies from Sri Lanka, which demonstrated marked factor and fibrinogen consumption followed by recovery after antivenom [13]. This is important comparative biology but indirect for *D. siamensis*. VICC should also not be used as a synonym for sepsis-associated disseminated intravascular coagulation. Bleeding, endothelial injury, fibrin generation, platelet changes, and renal microthrombi may coexist, but conventional coagulation tests do not measure all of them and their relative renal contributions remain unquantified.

A further distinction is required between systemic clotting-test recovery and the renal microenvironment. Normalization of a whole-blood clotting test or plasma fibrinogen may occur after endothelial activation, capillary injury, or intrarenal fibrin deposition has already initiated tissue damage. Conversely, severe VICC can occur without dialysis-requiring AKI. Serial coagulation markers are therefore evidence of venom pharmacodynamics and systemic risk, not a direct quantitative surrogate for renal microthrombotic burden.

### 5.2. Hemodynamic and Ischemic Injury

Hypotension, capillary leak, gastrointestinal or other bleeding, and reduced effective circulating volume can impair renal perfusion. In 52 Myanmar patients with renal failure, tubular injury markers and high renin concentrations supported renal ischemia and tubular damage [21]. Severe vasomotor disturbance may coexist with toxin-mediated changes inside the renal circulation. Once acute tubular injury or cortical necrosis is established, recovery may lag for days or weeks after circulation and coagulation improve. That lag is compatible with structural ischemic injury, but it does not exclude a direct toxic insult that occurred before venom was neutralized.

Renal autoregulation may be further challenged by vasoactive venom peptides, inflammatory mediators, and cardiac effects, although human *D. siamensis* data are insufficient to isolate these influences. Fluid resuscitation can restore macrocirculatory variables while medullary hypoxia or microvascular obstruction persists. For this reason, blood pressure at one time point and the absence of documented shock do not exclude an ischemic component.

### 5.3. Direct Tubular, Glomerular, and Inflammatory Effects

Human evidence for direct nephrotoxicity is suggestive rather than causal. Sixteen Myanmar patients without overt disseminated intravascular coagulation had abnormal urinary N-acetyl-beta-D-glucosaminidase or albumin excretion despite preserved conventional renal indices [22]. Histopathological series report tubular degeneration or necrosis, glomerular changes, interstitial lesions, vascular abnormalities, focal hemorrhage, and fibrin deposition [19,23]. No retained human or experimental *D. siamensis* study used venom-specific immunohistochemistry or immunofluorescence to localize an individual venom antigen within glomeruli, tubules, or renal microvessels. Conventional histology therefore describes injury patterns but does not identify a bound toxin.

Experimental evidence establishes biological plausibility. Whole Russell’s viper venom reduced filtration in an isolated perfused rat kidney [24]. In isolated rabbit kidneys, *D. siamensis* whole venom and PLA2 or metalloproteinase fractions altered renal hemodynamics and produced tubular injury, with pharmacologic evidence implicating platelet-activating factor [25]. Purified RVV-X rapidly impaired renal blood flow and filtration in rats while increasing coagulation markers [26]. This experiment is especially instructive because a purified venom factor affected the kidney through intravascular coagulation; it cannot be categorized cleanly as either direct or secondary.

Biochemical fractionation further implicates PLA2 and SVMP-rich fractions in renal injury [27]. Later rabbit studies link whole venom or fractions to oxidative and cytokine responses, membrane and ion-channel effects, and altered Bcl-2-family expression [28,29,30]. Reported exposures included purified components, isolated perfused kidneys, and in vivo or ex vivo rabbit models; doses, routes, and treatment timing differed from human bite exposure.

Across the experimental literature, renal endpoints included blood flow, glomerular filtration, tubular function, histologic injury, oxidative and cytokine markers, ion-channel responses, and apoptosis-related proteins [24,25,26,27,28,29,30]. No experiment compared all major toxin families at clinically matched circulating concentrations, and no human study measured the fraction of AKI attributable to a specific component.

### 5.4. Rhabdomyolysis, Hemolysis, and Pathway Interaction

Rhabdomyolysis and hemolysis can contribute through pigment nephropathy, oxidative stress, tubular obstruction, and vasoconstriction. In the mixed Yangon cohort, creatine kinase >500 IU/L was associated with AKI [10], while the Thai *D. siamensis* cohort found rhabdomyolysis associated with AKI and dialysis requirement [5]. These observational associations may partly indicate higher systemic venom exposure rather than an isolated mediator. Sri Lankan *D. russelii* data show that myotoxicity may be mild and geographically variable [14], so its magnitude should not be transferred directly to *D. siamensis*.

No human cohort simultaneously measured serial venom antigen, coagulation and endothelial markers, muscle or hemolysis markers, and kidney-injury biomarkers. The available studies therefore document co-occurring pathways but do not provide a causal mediation analysis or a quantitative ranking of mechanisms.

## 6. Antivenom: Product Matching, Timing, and Limits of Inference

An appropriate antivenom binds circulating venom components and can accelerate recovery of venom-driven coagulopathy. It cannot be expected to reverse completed tubular or cortical necrosis. Consequently, coagulation may normalize before creatinine without proving that the initial kidney insult was exclusively secondary. Persistent or recurrent coagulopathy may additionally reflect venom kinetics, venom load, delayed absorption, dose, and the neutralizing spectrum of the product.

Published antivenom studies used different endpoints. Human studies reported clotting recovery, AKI, dialysis, or renal severity [5,6,10,11], while experimental studies measured antivenomic binding, murine neutralization, biochemistry, histology, or renal function [3,4,12,15]. None was designed to compare serial venom-antigen clearance, coagulation recovery, standardized AKI stage, and long-term renal recovery in the same cohort.

Human timing data are observational. In the 1985 Myanmar cohort, antivenom restored blood coagulability but did not prevent every episode of renal failure, including among some patients treated within four hours [6]. In Taiwan, antivenom given three to six hours after systemic envenoming appeared to reduce renal severity relative to historical or delayed controls, but the report included only 13 nonrandomized patients [11]. In the mixed Yangon cohort, a delay of at least two hours was independently associated with AKI [10]. The 2026 Thai cohort found antivenom within two hours associated with lower odds of hemodialysis (odds ratio 0.153, 95% confidence interval 0.035–0.674) [5]. Residual confounding, referral and indication bias, uncertainty about the timing origin, and immortal-time effects prevent these associations from defining a universal cutoff.

In rats, Thai *D. siamensis* antivenom administered before or one hour after intravenous venom reduced biochemical and morphological nephrotoxicity at sufficient doses [15]. Antivenomic studies demonstrated cross-regional recognition within *D. siamensis*, but potency was not uniform and heterologous products performed less well in some experiments [3,4,12]. These designs tested early neutralization under controlled conditions rather than a human two-hour threshold.

Varespladib inhibited selected PLA2-mediated in vitro neurotoxic or myotoxic effects of Chinese *D. siamensis* venom [33]. The study did not assess human AKI prevention, kidney replacement therapy, or renal recovery.

## 7. Thrombotic Microangiopathy

Snakebite-associated TMA is characterized by microangiopathic hemolytic anemia, thrombocytopenia, and organ injury, often recognized with or after VICC. The strongest Russell’s-viper cohort evidence comes from Sri Lankan *D. russelii* and *Hypnale* envenoming, where TMA was associated with more prolonged AKI and greater dialysis requirements [34]. Systematic and practice-focused reviews found no convincing benefit of plasma exchange and no evidence that antivenom specifically prevents TMA [35,36]. These cohorts did not establish a *D. siamensis* frequency or treatment effect.

*D. siamensis*-specific TMA frequency is unknown. Historical cohorts did not systematically report serial blood films, lactate dehydrogenase, haptoglobin, bilirubin, and platelet trajectories [6,13,19,20,21,22,23], and contemporary cohorts did not apply a uniform prospective TMA definition [5,8,9,10,11].

Thrombocytopenia was common in systemic *D. siamensis* envenoming, but the retained studies did not consistently pair it with evidence of microangiopathic hemolysis and organ injury [2,5,6,8,9,10,11,13,19,20,21,22,23]. Consequently, the available literature cannot estimate the diagnostic yield of universal TMA screening.

## 8. Long-Term Renal Outcomes

No cohort provides a reliable *D. siamensis*-specific incidence of chronic kidney disease (CKD) after AKI. The earlier manuscript’s estimate of approximately 19% at 32 months could not be verified in the cited source and should not be retained. Available follow-up studies combine snake species and use different endpoints. A Sri Lankan descriptive series reported CKD at one year in 20 of 54 patients, but only 15 bites were attributed to Russell’s viper and 13 to *Hypnale* [37]. Indian cohorts reported composite adverse renal outcomes in 28.7% of 73 followed survivors and 48.8% of 43 followed survivors, respectively; mixed haemotoxic exposures, uncertain baseline kidney function, and loss to follow-up limit generalizability [38,39].

Conversely, a prospective authenticated Sri Lankan cohort found no incident CKD at one or four years among reviewed participants; reduced estimated glomerular filtration rate was attributed to pre-existing disease or comorbidity, although albuminuria was common [40]. Differences among studies include case mix, AKI severity, species, survivor and follow-up bias, baseline ascertainment, and outcome definition. No species-authenticated longitudinal *D. siamensis* cohort is available.

Existing follow-up studies variably reported persistent dysfunction, new CKD, albuminuria, or composite renal outcomes and did not uniformly account for death or loss to follow-up [37,38,39,40]. Their percentages cannot be relabeled as the long-term prognosis of *D. siamensis* envenoming.

## 9. Discussion

### 9.1. What Human Renal Evidence Is Specific to Daboia siamensis?

Species-authenticated and species-focused referral cohorts consistently show that AKI can be a major complication of systemic *D. siamensis* envenoming, sometimes requiring kidney replacement therapy [5,6,8,9,10,11,13,19,20,21,22,23]. They do not support a universal incidence because recruitment, species certainty, AKI definitions, referral severity, antivenom access, and dialysis practice differ. The Taiwanese rationale is therefore severity and clinical consequence rather than numerical dominance of this species among all snakebites [2,7]. Species documentation remains central to interpretation and product selection.

Reported associations with coagulopathy, shock, rhabdomyolysis, and antivenom delay are clinically useful for risk recognition but may also be index venom burden or referral severity [5,10]. Likewise, persistent kidney dysfunction after correction of coagulopathy confirms established injury but does not identify whether the initiating pathway was direct, hemodynamic, microvascular, pigment-related, or mixed.

### 9.2. Which Toxin Families and Systemic Responses Plausibly Contribute?

Proteomic, transcriptomic, and individual-component experiments support inclusion of PLA2, SVMPs including RVV-X, SVSPs, snaclecs, Kunitz inhibitors, and other components in a multifactorial model [3,4,12,16,17,18,24,25,26,27,28,29,30,32]. A purified venom factor can affect renal tissue while simultaneously activating coagulation, so ‘direct’ and ‘secondary’ mechanisms are not mutually exclusive. Toxins may injure renal cells or endothelium; bleeding, capillary leak, and shock may reduce perfusion; and muscle or red-cell injury may add pigment and oxidative burden. No human evidence ranks these pathways.

The individual-component models establish biological plausibility but not proportional causality in patients. Purified or intravenous exposure, isolated organs, species differences, and early treatment limit transfer. Selective neutralization, clinically realistic subcutaneous delivery, delayed treatment, blinded assessment, and matched concentrations would strengthen causal inference. The absence of *D. siamensis*-specific immunohistochemical or immunofluorescent localization of venom antigens in renal structures is a distinct evidence gap; conventional histology cannot substitute for toxin-specific localization.

### 9.3. What Can Be Concluded About Antivenom Timing and TMA?

Indicated, regionally appropriate antivenom should be administered as soon as feasible under local protocols because it neutralizes circulating venom and treats venom-driven coagulopathy [5,6,11,15,41]. Observational associations at two hours are vulnerable to timing error, residual confounding, referral bias, and indication bias; the preclinical studies used controlled early dosing. Current evidence therefore does not validate a universal two-hour renal-protection threshold. Coagulation recovery should not end renal surveillance because established structural injury can persist after circulating venom activity improves.

TMA evaluation should be targeted to a compatible trajectory: persistent or worsening thrombocytopenia, unexplained anemia or hemolysis, schistocytes, or AKI that is disproportionate or continues despite recovery from VICC and hemodynamic instability [34,35,36]. Thrombocytopenia alone is insufficient; bleeding, transfusion, infection, and other causes must be considered. Evidence does not support routine plasma exchange for snakebite-associated TMA without another established indication or a research protocol [35,36].

### 9.4. Can Long-Term Kidney Risk Be Quantified?

No *D. siamensis*-specific CKD incidence can be quantified. Mixed-species follow-up studies report conflicting outcomes and differ in baseline kidney ascertainment, AKI severity, endpoints, survival, and loss to follow-up [37,38,39,40]. Survivors of moderate or severe AKI should receive general post-AKI reassessment of kidney function, blood pressure, and albuminuria, including assessment at approximately three months where feasible [42]. This is an application of general AKI care, not a claim that *D. siamensis* has a known CKD conversion rate.

### 9.5. Evidence-Informed Clinical Pathway

Table 3 translates the evidence synthesis into a minimum clinical pathway. It is intentionally framed as an evidence-informed proposal rather than a validated *D. siamensis*-specific protocol; product, dose, repeat-dose criteria, coagulation testing, and referral pathways must follow regional guidance and available resources [5,6,11,15,35,36,41,42].

### 9.6. Future Directions

The key next step is a prospective, multicenter, species-authenticated cohort with prespecified parameters. Species certainty should use specimens or photographs when safe and validated venom antigen testing where available. Contemporary AKI staging, baseline creatinine assumptions, referral denominators, antivenom product/lot/dose/timing, dialysis indications, and three-month and one-year outcomes should be recorded.

Serial sampling should align venom antigen concentration with coagulation factors, endothelial and hemolysis markers, creatine kinase, urinalysis, and tubular or glomerular injury biomarkers. Venom samples should be linked to geography, snake age or size when known, proteomic or functional profiles, and the antivenom lot. Repeated-measures and prespecified causal models could distinguish severity predictors from mediators and test whether toxin clearance precedes pathway-specific recovery.

Renal tissue obtained for clinical indications or at autopsy should use harmonized pathology and, where validated reagents are available, toxin-specific immunohistochemistry, immunofluorescence, or complementary mass-spectrometric localization. Such work could test whether PLA2, RVV-X/SVMP, or other antigens localize to glomerular, tubular, endothelial, or microvascular structures. Experimental studies should use realistic subcutaneous exposure, delayed antivenom, selective neutralization, blinding, and renal-relevant endpoints.

*D. siamensis*-specific TMA studies require prespecified hematologic definitions and serial sampling. Long-term studies should distinguish nonrecovery by 90 days, new CKD, recurrent AKI, albuminuria, and composite outcomes while accounting for death and loss to follow-up. Adjunct toxin inhibitors should progress only after target engagement, safety, pharmacokinetics, and incremental benefit over regional antivenom are established.

### 9.7. Limitations by Review Question

For question 1, human *D. siamensis* reports are few, geographically concentrated, referral-based, and heterogeneous in species confirmation, AKI definitions, and treatment access; this precludes pooled incidence. For question 2, venom-analytical and animal studies resolve components and pathways but use doses, routes, hosts, and endpoints that do not quantify their contribution to human AKI; renal venom-antigen localization is absent. For question 3, antivenom timing is observational and TMA evidence is predominantly indirect, so no universal time cutoff, screening strategy, or plasma-exchange benefit can be inferred. For question 4, no species-authenticated longitudinal cohort quantifies *D. siamensis*-specific CKD risk.

At the review-process level, screening and appraisal were not independently duplicated, selective interpretation remains possible, and OpenAlex broadened retrieval at the cost of low-relevance records. Publication bias is plausible because severe renal presentations and positive mechanistic experiments are more likely to be reported than uncomplicated bites or neutral findings. These limitations preclude causal ranking, universal treatment thresholds, or direct transfer of *D. russelii* estimates to *D. siamensis*.

## 10. Conclusions

AKI after *D. siamensis* envenoming is a multifactorial toxin-mediated syndrome. Venom proteins can affect renal cells, coagulation, endothelium, perfusion, muscle, red cells, and inflammation; human evidence does not establish that one route predominates. Clinicians must maintain renal vigilance and serial renal/coagulation surveillance irrespective of whether injury is direct, indirect, or mixed. Regionally appropriate antivenom should be administered promptly when indicated, without converting observational timing associations into a rigid two-hour cutoff. TMA evaluation should be targeted, and routine plasma exchange is unsupported without another established indication or a research protocol. *D. siamensis*-specific CKD risk remains unknown. Prospective species-authenticated studies linking venom kinetics and toxin profiles to standardized renal phenotypes and follow-up are required.

## Figures and Tables

**Table 1:** Species-relevant human clinical and pathological evidence for kidney injury after *Daboia siamensis* envenoming.

Study and Setting	Species Ascertainment	Renal or Treatment Finding	Principal Limitation	Confidence for *D. siamensis* Inference
Myint-Lwin et al., 1985 [6]; Myanmar; clinical cohort, *n* = 123	Proved bites; historical *V. russelli siamensis* assignment	Oliguria accompanied systemic envenoming; antivenom corrected coagulability but did not prevent all renal failure	Pre-KDIGO definition, historical care, and no randomized timing comparison	Moderate
Than-Than et al., 1989 [19]; Myanmar; fatal autopsy series, *n* = 3	Clinical/venom-antigen context in *D. siamensis* range	Focal hemorrhage, fibrin deposition, shock, and acute tubular necrosis	Extreme severity selection; pathology cannot apportion competing mechanisms	Low
Tin-Nu-Swe et al., 1993 [21]; Myanmar; renal-failure series, *n* = 52	Historical *D. russelii* siamensis assignment	Tubular protein markers and high renin supported tubular injury and ischemia	No non-AKI comparator; renal definition predates KDIGO	Low–moderate
Han et al., 1993 [20]; Myanmar; severe-defibrination series, *n* = 20	Proven bites; historical *D. russelii* siamensis assignment	Urinary fibrin degradation products were higher in 10 renal-failure cases	Small sample and confounding by overall severity	Low
Win-Aung et al., 1998 [22]; Myanmar; series without overt DIC, *n* = 16	Historical *D. russelii* siamensis assignment	Tubular/albumin markers suggested renal stress without overt DIC	Subclinical hemostatic, endothelial, and perfusion effects not excluded	Low
Hung et al., 2002 [2]; Taiwan; retrospective series, *n* = 18	Regional and clinical assignment	Renal failure, defibrination, bleeding, hemolysis, and systemic thrombosis described	Rare-event referral series; no incidence denominator; historical era	Low
Hung et al., 2006 [11]; Taiwan; serum-venom case series, *n* = 13	Serum venom detected plus regional context	Earlier antivenom appeared associated with lower renal severity	Very small, nonrandomized historical/delayed comparison	Low–moderate
Aye et al., 2017 [10]; Myanmar; prospective mixed-snakebite cohort, *n* = 258	147/174 identified snakes were Russell’s vipers; species not separable in all analyses	AKI 54.3%; systemic variables and antivenom delay ≥2 h associated with AKI	Species-mixed adjusted model, referral effects, and timing confounding	Moderate for triage; low for species-specific effects
White et al., 2019 [8] and Alfred et al., 2019 [9]; Mandalay registry	Specimen, clinician assessment, or patient recognition; presumed *D. siamensis*	Registry AKI 59.8% overall; companion analysis reported AKI 71% and dialysis 31% among 686 presumed bites	Overlapping reports, tertiary referral severity, pragmatic AKI definition, and local dialysis access	Moderate for local burden; low for general incidence
Tansuwannarat et al., 2026 [5]; Thailand; retrospective poison-center study, *n* = 185	101 definite by specimen/photo; remaining cases clinically diagnosed	AKI 50.3%; antivenom ≤2 h associated with lower odds of hemodialysis	Referral selection, retrospective timing, residual confounding; association is not a cutoff	Moderate

Raw proportions are presented only within their original study context and should not be compared as incidence estimates. DIC: disseminated intravascular coagulation; RRT: renal replacement therapy.

**Table 2:** Toxin/component evidence map for *Daboia siamensis*-associated kidney injury.

Toxin/Component Family	*D. siamensis* Evidence/Model	Plausible Renal Pathway	Antivenom/Translation Implication	Critical Limitation
Phospholipase A2 (PLA2)	22.2–48.37% across regional proteomes; purified PLA2 and PLA2-rich fractions caused renal functional and structural effects in rabbit in vivo/ex vivo models [3,4,25,27,29]	Membrane injury, inflammation, altered renal hemodynamics, myotoxicity, and pigment burden	Neutralization may be product- and dose-dependent; adjunct PLA2 inhibition remains experimental	Purified or fraction doses do not quantify the human contribution
SVMPs and factor X activator (RVV-X)	Approximately 2–3% in Thai/Indonesian proteomes; purified RVV-X altered renal flow, filtration, and coagulation in rats; SVMP-rich fractions injured rabbit kidneys [4,26,27,32]	Basement-membrane/endothelial injury, hemorrhage, intrarenal coagulation, and tubular stress	Relevant proteoforms and coagulant activity must be neutralized	Purified intravenous exposure differs from a human bite
SVSPs and procoagulant network	Approximately 13–23% in Thai/Indonesian proteomes; multiple transcripts and ontogenetic variation [4,16,17,18]	Consumption coagulopathy, fibrin generation, bleeding, and microvascular disturbance	Coagulation recovery is a pharmacodynamic endpoint, not proof of renal recovery	No purified *D. siamensis* SVSP renal model; human attribution is unresolved
Snaclecs and disintegrins	Regional proteomic variation and multiple transcripts/isoforms [3,4,17]	Platelet activation or inhibition, adhesion changes, and endothelial–hemostatic interaction	Antivenomic binding does not establish functional neutralization	Renal-specific causal evidence is sparse
LAAO and oxidative/inflammatory components	Regional abundance varies; whole venom/fractions induce oxidative and cytokine responses [4,28]	Oxidative stress, inflammatory amplification, endothelial and tubular injury	Potential biomarker or adjunct target, not an established treatment indication	Pathway markers may be downstream rather than causal
Kunitz inhibitors, BPP-CNP precursors, and other proteins	Kunitz inhibitors comprised 22.4–28.2% in Thai, Guangxi, and Taiwan venoms but were negligible in the Indonesian sample; broader repertoires occur in omic studies [3,4,16,17]	Possible modulation of proteases, vascular tone, permeability, and toxin distribution	May influence regional phenotype or product coverage	No purified Kunitz renal model; detection does not establish toxicity
Whole-venom mixture	Human syndromes and in vivo/ex vivo studies integrate multiple components [15,18,25]	Interacting cellular, coagulation, endothelial, perfusion, pigment, and inflammatory pathways	Clinically relevant antivenom and delayed-treatment models should target the mixture	Mixture effects prevent attribution to one family

The table maps evidence and plausible pathways; it does not rank causal importance in humans. BPP-CNP: bradykinin-potentiating peptide/C-type natriuretic peptide; LAAO: L-amino acid oxidase; PLA2: phospholipase A2; SVMP: snake venom metalloproteinase; SVSP: snake venom serine protease.

**Table 3:** Proposed evidence-informed clinical pathway for renal and coagulation care after *Daboia siamensis* envenoming.

Phase	Minimum Assessment and Action	Evidence Boundary
Presentation	Stabilize airway, breathing, and circulation; document bite time, transfer history, species certainty, and prior antivenom. Record urine output; obtain creatinine/electrolytes, urinalysis, complete blood count, and locally appropriate coagulation testing; add creatine kinase and hemolysis tests when indicated.	Do not delay resuscitation or indicated antivenom while awaiting complete testing.
Specific antivenom	Give the indicated, regionally appropriate product promptly according to local dose and repeat-dose criteria; document product, lot, dose, and timing.	No universal two-hour renal-protection cutoff is validated; neutralization varies by venom origin and product.
Serial monitoring	Repeat clinical perfusion, limb/neurovascular examination, urine output, creatinine/electrolytes, blood count, and coagulation tests according to severity and local protocol.	Clotting recovery does not prove renal recovery or exclude established structural injury.
AKI or deterioration	Optimize perfusion, avoid additional nephrotoxins, correct electrolyte/acid-base disturbances, seek nephrology/critical-care support, and initiate kidney replacement therapy for standard clinical indications.	No venom-specific creatinine, creatine kinase, or dialysis threshold is validated.
Suspected TMA	If thrombocytopenia persists/worsens with unexplained anemia, hemolysis, schistocytes, or disproportionate/progressive AKI, assess blood film, lactate dehydrogenase, bilirubin, haptoglobin, and alternative causes.	Routine universal screening and routine plasma exchange are not supported; use another established indication or research protocol.
Discharge and follow-up	Document renal recovery, blood pressure, creatinine/eGFR, and albuminuria after significant AKI, including reassessment at approximately three months where feasible.	*D. siamensis*-specific CKD incidence is unknown; follow general post-AKI principles.

This pathway has not been prospectively validated. Antivenom product, dose, repeat dosing, coagulation testing, supportive care, and referral must follow local or national protocols. eGFR: estimated glomerular filtration rate; TMA: thrombotic microangiopathy.

## Data Availability

No new datasets were created or analyzed. Reproducible search strategies, result accounting, and evidence appraisals are provided in the Supplementary Material.

## Supplementary Materials

The following supporting information can be downloaded at: https://www.mdpi.com/article/10.3390/toxins18090389/s1, Table S1: Reproducible search strategies and result accounting; Figure S1: Structured evidence-identification and curation flow. Screening and appraisal were not performed independently by two reviewers; therefore, the flow is descriptive and is not presented as a PRISMA flow diagram; Table S2: Human evidence appraisal; Table S3: Venomic and experimental evidence appraisal; and Table S4: Evidence-transferability matrix.

## Abbreviations

The following abbreviations are used in this manuscript:

AKI	Acute kidney injury
AKIN	Acute Kidney Injury Network
BPP-CNP	Bradykinin-potentiating peptide/C-type natriuretic peptide
CKD	Chronic kidney disease
DIC	Disseminated intravascular coagulation
eGFR	estimated glomerular filtration rate
KDIGO	Kidney Disease: Improving Global Outcomes
KRT	Kidney replacement therapy
LAAO	L-amino acid oxidase
PLA2	Phospholipase A2
RVV-X	Russell’s viper venom factor X activator
SVMP	Snake venom metalloproteinase
SVSP	Snake venom serine protease
TMA	Thrombotic microangiopathy
VICC	Venom-induced consumption coagulopathy
