# Supplementary material for: From Venom to Kidney Injury: A Critical Review of Daboia siamensis Envenoming
Source: Toxins (Basel). 2026 Sep 9;18(9):389. doi: 10.3390/toxins18090389 (PMC13611550; doi:10.3390/toxins18090389)
Supplement: Supplementary file 1 [file toxins-18-00389-s001.zip › toxins-4544541-supplementary.pdf]

toxins

MDPI

Supplementary Materials: From Venom to Kidney Injury: A Critical Review of *Daboia siamensis* Envenoming

Ren-Chieh Wu and Kuang-Ting Yeh

Table S1. Reproducible search strategies and result accounting.

Table S1a. Exact PubMed search blocks run on 3 August 2026.

Search block	Exact PubMed query	Records
Q1: *D. siamensis* renal/treatment	(("*Daboia siamensis*"[Title/Abstract] OR "*Vipera russelli siamensis*"[Title/Abstract] OR "*Vipera russelii siamensis*"[Title/Abstract] OR "*Daboia russelli siamensis*"[Title/Abstract] OR "*Daboia russelii* siamensis"[Title/Abstract] OR "eastern Russell's viper"[Title/Abstract] OR "Siamese Russell's viper"[Title/Abstract] OR "Formosan Russell's viper"[Title/Abstract]) AND ("acute kidney injury"[Title/Abstract] OR "acute renal failure"[Title/Abstract] OR kidney[Title/Abstract] OR renal[Title/Abstract] OR nephrotox*[Title/Abstract] OR dialysis[Title/Abstract] OR coagulopath*[Title/Abstract] OR "thrombotic microangiopathy"[Title/Abstract] OR antivenom[Title/Abstract])) AND ("1900/01/01"[Date - Publication] : "2026/08/03"[Date - Publication])	65
Q2: *D. russelii* renal/treatment	(("*Daboia russelii*"[Title/Abstract] OR "*Daboia russelli*"[Title/Abstract] OR "*Vipera russelli*"[Title/Abstract] OR "western Russell's viper"[Title/Abstract] OR "Sri Lankan Russell's viper"[Title/Abstract] OR "Indian Russell's viper"[Title/Abstract]) AND ("acute kidney injury"[Title/Abstract] OR "acute renal failure"[Title/Abstract] OR kidney[Title/Abstract] OR renal[Title/Abstract] OR nephrotox*[Title/Abstract] OR dialysis[Title/Abstract] OR coagulopath*[Title/Abstract] OR "thrombotic microangiopathy"[Title/Abstract] OR antivenom[Title/Abstract])) AND ("1900/01/01"[Date - Publication] : "2026/08/03"[Date - Publication])	196
Q3: Long-term renal outcomes	((snakebite[Title/Abstract] OR "snake envenomation"[Title/Abstract] OR "snake envenoming"[Title/Abstract]) AND ("long-term renal"[Title/Abstract] OR "long term renal"[Title/Abstract] OR "chronic kidney disease"[Title/Abstract] OR CKD[Title/Abstract] OR "renal outcome"[Title/Abstract] OR "kidney outcome"[Title/Abstract])) AND ("1900/01/01"[Date - Publication] : "2026/08/03"[Date - Publication])	34
Q4: *D. siamensis* venomics/antivenomics	(("*Daboia siamensis*"[Title/Abstract] OR "*Vipera russelli siamensis*"[Title/Abstract] OR "*Vipera russelii siamensis*"[Title/Abstract] OR "*Daboia russelli siamensis*"[Title/Abstract] OR "*Daboia russelii* siamensis"[Title/Abstract] OR "eastern Russell's viper"[Title/Abstract] OR "Siamese Russell's viper"[Title/Abstract] OR "Formosan Russell's viper"[Title/Abstract]) AND (venom*[Title/Abstract] OR proteom*[Title/Abstract] OR transcriptom*[Title/Abstract] OR antivenom*[Title/Abstract] OR neutraliz*[Title/Abstract] OR phospholipase[Title/Abstract] OR metalloproteinase[Title/Abstract] OR "factor X activator"[Title/Abstract] OR toxin*[Title/Abstract])) AND ("1900/01/01"[Date - Publication] : "2026/08/03"[Date - Publication])	100

PubMed accounting: 395 records across queries; 88 cross-query duplicates; 307 unique PubMed records.

Table S1b. OpenAlex title-and-abstract search blocks run on 3 August 2026.		
**Search block**	**Exact OpenAlex query**	**Records**
Q1: *D. siamensis* renal/treatment	("*Daboia siamensis*" OR "*Vipera russelli siamensis*" OR "*Vipera russelii siamensis*" OR "*Daboia russelli siamensis*" OR "*Daboia russelii* siamensis" OR "eastern Russell's viper" OR "Siamese Russell's viper" OR "Formosan Russell's viper") AND ("acute kidney injury" OR "acute renal failure" OR kidney OR renal OR nephrotoxicity OR dialysis OR coagulopathy OR "thrombotic microangiopathy" OR antivenom); fields: title_and_abstract.search; date filter 1900-01-01 to 2026-08-03	91
Q2: *D. russelii* renal/treatment	("*Daboia russelii*" OR "*Daboia russelli*" OR "*Vipera russelli*" OR "western Russell's viper" OR "Sri Lankan Russell's viper" OR "Indian Russell's viper") AND ("acute kidney injury" OR "acute renal failure" OR kidney OR renal OR nephrotoxicity OR dialysis OR coagulopathy OR "thrombotic microangiopathy" OR antivenom); fields: title_and_abstract.search; date filter 1900-01-01 to 2026-08-03	326
Q3: Long-term renal outcomes	(snakebite OR "snake envenomation" OR "snake envenoming") AND ("long-term renal" OR "long term renal" OR "chronic kidney disease" OR "renal outcome" OR "kidney outcome"); fields: title_and_abstract.search; date filter 1900-01-01 to 2026-08-03	63
Q4: *D. siamensis* venomics/antivenomics	("*Daboia siamensis*" OR "*Vipera russelli siamensis*" OR "*Vipera russelii siamensis*" OR "*Daboia russelli siamensis*" OR "*Daboia russelii* siamensis" OR "eastern Russell's viper" OR "Siamese Russell's viper" OR "Formosan Russell's viper") AND (venom OR venomics OR proteomics OR transcriptomics OR antivenom OR antivenomics OR neutralization OR phospholipase OR metalloproteinase OR "factor X activator" OR toxin); fields: title_and_abstract.search; date filter 1900-01-01 to 2026-08-03	158

OpenAlex accounting: 638 records across queries; 121 cross-query duplicates; 517 unique OpenAlex records. Cross-source deduplication left 534 unique records; 227 OpenAlex records were not duplicates of PubMed records.

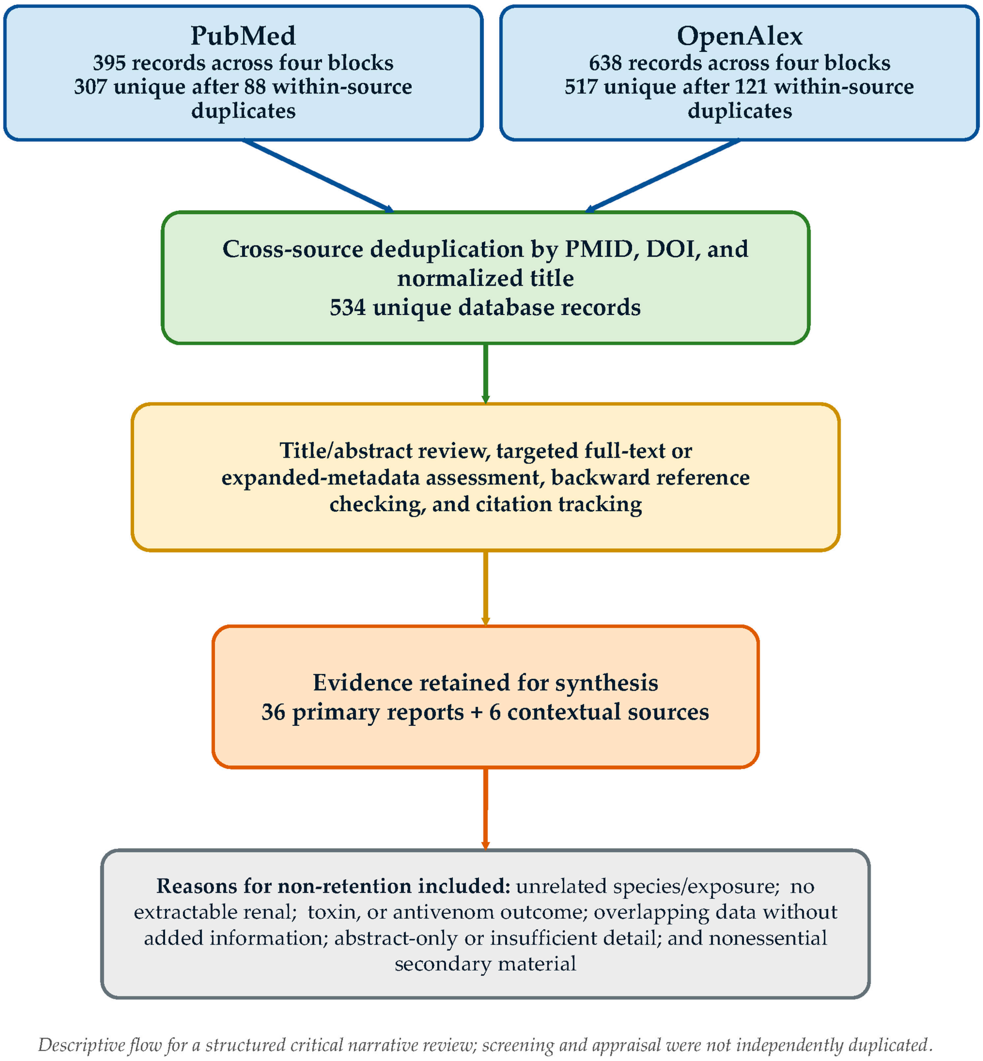

Figure S1. Structured evidence-identification and curation flow. Screening and appraisal were not performed independently by two reviewers; therefore, the flow is descriptive and is not presented as a PRISMA flow diagram.

Table S2. Human evidence appraisal.

Study	Design	Species certainty	Outcome definition	Confounding/comparator	Follow-up	Overall confidence/applicability
Myint-Lwin et al., 1985 [6]	Clinical cohort	Clear historical assignment	Pre-KDIGO/oliguria	Limited	In-hospital	Moderate
Than-Than et al., 1989 [19]	Fatal autopsy series	Venom-antigen/clinical context	Pathology	No living comparator	Fatal cases	Low
Tin-Nu-Swe et al., 1993 [21]	AKI series	Clear historical assignment	Creatinine/biomarkers	No non-AKI comparator	Acute	Low–moderate
Han et al., 1993 [20]	Small comparative series	Proven bites	Historical renal failure	Severity confounding	Acute	Low
Win-Aung et al., 1998 [22]	Series without overt DIC	Clear historical assignment	Urinary biomarkers	No controls; subclinical pathways unresolved	5 days	Low
Hung et al., 2002 [2]	Retrospective case series	Regional/clinical	Variable renal failure	No denominator or control	Hospital course	Low
Hung et al., 2006 [11]	Serum-venom case series	Serum venom plus region	Renal severity; no modern staging	Historical/delayed comparator	Hospital course	Low–moderate
Aye et al., 2017 [10]	Prospective mixed cohort	Mixed; 147/174 identified were Russell's vipers	AKIN staging	Adjusted model; residual confounding	In-hospital	Moderate overall; low species applicability
White et al., 2019 [8]	Prospective hospital registry	Mixed identification methods	Registry AKI/RRT	Referral bias; no community denominator	In-hospital	Moderate local; low transportability
Alfred et al., 2019 [9]	Companion analysis of Mandalay registry	Presumed *D. siamensis*	Pragmatic AKI composite	Overlaps White 2019; referral and treatment-access effects	In-hospital	Moderate local; not independent
Tansuwannarat et al., 2026 [5]	Retrospective poison-center cohort	101/185 definite	AKI and hemodialysis	Multivariable predictors; timing/referral bias remains	In-hospital	Moderate
Isbister et al., 2015 [13]	Prospective *D. russelii* cohort	Sri Lankan *D. russelii*	Coagulation kinetics	Strong serial measurement	48 h	Higher for VICC; indirect species

Study	Design	Species certainty	Outcome definition	Confounding/comparator	Follow-up	Overall confidence/applicability
Wijewickrama et al., 2020 [34]	Prospective AKI cohort	*D. russelii* plus *Hypnale*	Prespecified TMA; renal outcome	Treatment not randomized	3 months	Moderate; indirect species
Herath et al., 2012 [37]	Retrospective follow-up	Mixed; 15 Russell's viper	CKD at 1 year	Selection/baseline uncertainty	1 year	Low
Priyamvada et al., 2020 [38]	Prospective haemotoxic AKI cohort	Mixed/unspecified species	KDIGO; composite renal outcome	73/144 survivors followed	To ~2.5 years	Moderate; indirect
Kumar et al., 2022 [39]	Retrospective AKI cohort	Mixed/unspecified species	AKI/composite renal outcome	43 survivors attended follow-up	Mean 30 months	Low–moderate; indirect
Waiddyanatha et al., 2023 [40]	Prospective authenticated cohort	Mixed Sri Lankan species	eGFR and albuminuria	Comorbidity considered; few AKI cases	1 and 4 years	Higher for cohort; indirect

Appraisal rule: design-appropriate domains considered clear inclusion, reliable exposure/species classification, valid outcome measurement, appropriate comparator or confounding control, and complete follow-up. 'Higher' required most domains to be met without a critical applicability concern; 'moderate' indicated at least one material limitation; 'low' indicated small or selected samples, unclear definitions, or major confounding. No numeric summary score was used.

Table S3. Venomic and experimental evidence appraisal.

Study	Evidence type/model	Main contribution	Key risk of bias/applicability issue	Confidence
Tan et al., 2018 [3]	Regional proteomics/neutralization	Defines dominant families and Guangxi–Taiwan antivenom cross-neutralization	Pooled venom; experimental endpoints do not establish renal clinical efficacy	Moderate analytical
Yee et al., 2018 [32]	Myanmar venom-gland transcriptomics	Characterizes diverse SVMP transcripts including RVV-X-related sequences	Transcript abundance is not secreted dose or renal causality	Moderate analytical
Lingam et al., 2019 [12]	Immunoreactivity/murine neutralization	Thai antivenom neutralized Thai and Indonesian venoms	Short report; nonrenal experimental endpoints	Moderate preclinical
Lingam et al., 2020 [4]	Thai/Indonesian proteomics and immunoprofiling	Shows conserved families plus regional composition/recognition differences	Pooled venoms; binding is not identical to clinical neutralization	Moderate analytical
Saethang et al., 2022 [16]	Integrated genome/transcriptome/proteome	Expands toxin repertoire and isoform resolution	Functional renal relevance of many components untested	Moderate analytical
Yee et al., 2023 [17]	Myanmar venom-gland transcriptomics	Identifies 23 toxin-gene families and 53 full-length transcripts	Expression, secretion, dose, and renal causality differ	Moderate analytical
Ratcliffe et al., 1989 [24]	Isolated perfused rat kidney; whole venom	Direct organ-level functional effect	Systemic syndrome absent; allocation/blinding incompletely reported	Moderate plausibility; low clinical transfer
Suntravat et al., 2011 [26]	Rat; purified RVV-X	Links factor X activation, coagulation markers, and renal hemodynamics	Purified intravenous exposure	Moderate mechanistic; low quantitative transfer
Chaiyabutr et al., 2020 [25]	Isolated perfused rabbit kidney; venom/fractions	Renal function, histology, and pathway antagonist	Ex vivo; systemic VICC/hemodynamics absent	Moderate mechanistic
Chaisakul et al., 2021 [27]	Biochemical plus in vivo/ex vivo fractions	PLA2/SVMP characterization with renal injury	Fraction doses differ from human exposure	Moderate mechanistic
Chaiyabutr et al., 2022 [18]	Rabbit; age-stratified pooled venoms	Links ontogenetic composition to renal effects	Animal model; does not prove human age-risk gradient	Moderate mechanistic
Chaiyabutr et al., 2024 [28]	Rabbit in vivo/ex vivo	Oxidative and cytokine pathways	Translation and pathway specificity uncertain	Moderate mechanistic
Chaiyabutr et al., 2025 [29]	Isolated rabbit kidney; PLA2	Membrane/ion-channel-related renal effects	Purified fraction and ex vivo model	Moderate mechanistic
Chaiyabutr et al., 2026 [30]	Rabbit in vivo/ex vivo; venom/fractions	Bcl-2-family response supports apoptotic stress	Preclinical exposure and reporting limitations	Moderate mechanistic
Chaisakul et al., 2019 [15]	Rat nephrotoxicity plus antivenom	Geographic neutralization and early high-dose renal protection	Intravenous venom; pretreatment/1 h; no human cutoff inference	Moderate preclinical; low threshold transfer

Study	Evidence type/model	Main contribution	Key risk of bias/applicability issue	Confidence
Lay et al., 2023 [33]	In vitro antivenom/varespladib	Tests product and PLA2 inhibitor against Chinese venom toxicity	Neuro/myotoxic, not renal or clinical endpoints	Moderate target evidence; low renal transfer

Appraisal rule: analytical studies were considered for venom-source definition, replication, proteomic/transcriptomic identification, functional validation, and relevance to renal or antivenom questions. Experimental studies were considered for controls, allocation/blinding reporting, outcome completeness, exposure relevance, and treatment timing. Mechanistic confidence and direct clinical transferability were rated separately.

Table S4. Evidence-transferability matrix.

Domain	*D. siamensis*-specific evidence	Indirect evidence	Defensible conclusion
AKI frequency	Referral cohorts report frequent AKI among hospitalized/systemically envenomed patients [5,8–10]	Cross-snakebite pooled estimates are highly heterogeneous [31]	No universal incidence, dialysis, or mortality rate
Direct renal toxicity	Isolated-kidney and animal models show tubular, hemodynamic, oxidative, inflammatory, and apoptotic effects [24,25,27–30]	Histology after other viper bites is not mechanism-specific	Biological plausibility is established preclinically; the proportional human contribution is unknown
VICC/microvascular injury	Human reports show defibrination, fibrin deposition, shock, and renal injury [6,19,20]	Detailed kinetics and much TMA evidence come from *D. russelii*/*Hypnale* [13,34]	Systemic hemostatic injury is important; *D. russelii* TMA frequency cannot be transferred
Antivenom timing	Small series and observational cohorts support prompt therapy [5,10,11]	Preclinical renal protection used early, high-dose treatment [15]	Give indicated regional antivenom as soon as feasible; no proven universal one- or two-hour cutoff [41]
TMA evaluation	*D. siamensis* frequency is unknown because systematic testing is sparse	*D. russelii*/*Hypnale* cohorts and reviews define the phenotype and show no proven plasma-exchange benefit [34–36]	Use targeted testing for compatible hematologic or renal findings
Long-term outcomes	No species-authenticated longitudinal *D. siamensis* cohort	Mixed-species studies have conflicting outcomes [37–40]	*D. siamensis*-specific CKD incidence is unknown; apply general post-AKI follow-up
Renal venom-antigen localization	No retained *D. siamensis* study used toxin-specific immunohistochemistry or immunofluorescence to localize antigen in glomeruli, tubules, or renal microvessels	Planted-antigen reports from other venomous animals are not species-transferable	Direct renal localization remains an explicit evidence gap; conventional histology cannot identify a bound toxin

D. russelii, mixed-species, and experimental evidence was used to frame hypotheses and clinical caution, not to generate *D. siamensis*-specific incidence or treatment-effect estimates.
